# Is universal health coverage the practical expression of the right to health care?

**DOI:** 10.1186/1472-698X-14-3

**Published:** 2014-02-24

**Authors:** Gorik Ooms, Laila A Latif, Attiya Waris, Claire E Brolan, Rachel Hammonds, Eric A Friedman, Moses Mulumba, Lisa Forman

**Affiliations:** 1 Department of Public Health, Institute of Tropical Medicine, Nationalestraat 155, 2000 Antwerpen, Belgium; 2 Law and Development Research Group, Faculty of Law, University of Antwerp, Venusstraat 23, 2000 Antwerpen, Belgium; 3 O’Neill Institute for National and Global Health Law, Georgetown University Law Center, 600 New Jersey Avenue, NW, Washington, DC 20001 USA; 4 Rachier & Amollo Advocates, 5th Floor Mayfair Centre, Ralph Bunche Road, P. O. Box 55645–00200, Nairobi, Kenya; 5 Commercial Law Department, School of Law, University of Nairobi, Parklands Campus, P. O. Box 30197 – 00100, Nairobi, Kenya; 6 School of Population Health, Faculty of Medicine and Biomedical Sciences, University of Queensland, Herston Road, Herston, QLD 4006, Australia; 7 Center for Health, Human Rights and Development, Plot 614 Tufnell Drive, Kamwokya – Kampala, P.O Box 16617, Wandegeya Kampala, Uganda; 8 Dalla Lana School of Public Health, University of Toronto, 155 College Street, Toronto, ON M5T3M7, Canada

**Keywords:** Millennium development goals, Universal health coverage, Right to health

## Abstract

The present Millennium Development Goals are set to expire in 2015 and their next iteration is now being discussed within the international community. With regards to health, the World Health Organization proposes universal health coverage as a ‘single overarching health goal’ for the next iteration of the Millennium Development Goals.

The present Millennium Development Goals have been criticised for being ‘duplicative’ or even ‘competing alternatives’ to international human rights law. The question then arises, if universal health coverage would indeed become the single overarching health goal, replacing the present health-related Millennium Development Goals, would that be more consistent with the right to health? The World Health Organization seems to have anticipated the question, as it labels universal health coverage as “by definition, a practical expression of the concern for health equity and the right to health”.

Rather than waiting for the negotiations to unfold, we thought it would be useful to verify this contention, using a comparative normative analysis. We found that – to be a practical expression of the right to health – at least one element is missing in present authoritative definitions of universal health coverage: a straightforward confirmation that international assistance is essential, not optional.

But universal health coverage is a ‘work in progress’. A recent proposal by the United Nations Sustainable Development Solutions Network proposed universal health coverage with a set of targets, including a target for international assistance, which would turn universal health coverage into a practical expression of the right to health care.

## Correspondence/Findings

### Background

The present Millennium Development Goals (MDGs) can be seen as a “super-norm of ending global poverty” [1]. Some would argue that this super-norm was not needed, as there was one already, in article 28 of the Universal Declaration of Human Rights: “Everyone is entitled to a social and international order in which the rights and freedoms set forth in this Declaration can be fully realized” [2]. Opinions about the added value of the MDGs to the relevant prescriptions of international human rights law vary, from ‘consistent’ and ‘complementary’ , to ‘not necessarily inconsistent’ and ‘duplicative’ , to ‘competing alternatives’ [3].

The present MDGs are set to expire in 2015 and their next iteration is now being discussed within the international community. With regards to health, the World Health Organization (WHO) proposes the concept of universal health coverage (UHC) as a “single overarching health goal” for the next iteration of the MDGs [4]. Whether this proposal will be accepted remains to be seen, but the adoption in December 2012 of a United Nations General Assembly (UNGA) resolution in favour of UHC increases the prominence of UHC in the post-MDG debate [5], the High-Level Panel of Eminent Persons on the Post-2015 Development Agenda mentions UHC [6], and the Sustainable Development Solutions Network gave UHC a prominent place in its proposal for Sustainable Development Goals [7].

The question then arises, if UHC would indeed become the single overarching health goal, replacing the present health-related MDGs – the expression ‘health-related MDGs’ usually refers to MDG4 (child mortality), MDG5 (maternal health), and MDG6 (HIV/AIDS, malaria and other diseases) – would that be consistent with the right to health? The WHO seems to have anticipated the question, as it considers UHC “by definition, a practical expression of the concern for **health equity and the right to health**” (emphasis in original) [4].

To verify the WHO contention on UHC being the practical expression of the right to health, we conducted a comparative normative analysis [8]. If UHC became the new ‘single overarching’ health goal, with the political normative power the MDGs do seem to have, what can people expect this goal to achieve, and would that outcome really provide the practical or political translation of what people can legitimately expect from the right to health?

## Methods

Many scholars and global health practitioners express their opinions about what the right to health or UHC entails. Although these opinions are valuable, they do not carry authoritative weight. If we want to compare the normative content of the right to health with the normative content of UHC, we have to agree first on the authoritative sources.

With regards to the right to health, the authoritative sources are fairly easily identifiable. The human rights mentioned in the Universal Declaration of Human Rights (UDHR) was further elaborated in two primary covenants that together with the UDHR make up the International Bill of Rights: the International Covenant on Civil and Political Rights [9], and the International Covenant on Economic, Social and Cultural Rights [10]. The right to health is included in the International Covenant on Economic, Social and Cultural Rights (International Covenant) [10]. To monitor compliance with the International Covenant, a Committee on Economic, Social and Cultural Rights (Committee) was created, which issues authoritative interpretations of rights included in the International Covenant, the so-called ‘general comments’. General Comment 14 clarifies the scope and the content of the right to health [11].

With regards to UHC, authoritative sources are less easy to identify. There are no treaties about UHC. The 2005 World Health Assembly resolution on “Sustainable health financing, universal coverage and social health insurance” (2005 WHA Resolution) [12], has been accepted by the World Health Assembly, where almost all countries are represented and each country has one vote. We can therefore consider it as similar to a declaration by the United Nations. The same can be argued about the United Nations General Assembly resolution on “Global health and foreign policy”, adopted in December 2012 (2012 UNGA Resolution) [5]. But both resolutions are relatively short and may not fully reflect the concept of UHC that the WHO has in mind when proposing it as the ‘single overarching’ new health goal. As there is no real equivalent of a general comment for UHC, we decided to include the 2010 World Health Report on UHC (2010 World Health Report) [13], and the WHO discussion paper on health in the post-2015 agenda (2012 WHO Discussion Paper) [4] in our analysis as well, assuming that they are both approved by the leadership of the WHO.

We faced an additional problem because the right to health is, according to General Comment 14, “an inclusive right extending not only to timely and appropriate health care but also to the underlying determinants of health, such as access to safe and potable water and adequate sanitation, an adequate supply of safe food, nutrition and housing, healthy occupational and environmental conditions, and access to health-related education and information, including on sexual and reproductive health” [9], whereas UHC is focused on health care – including “prevention, promotion, treatment and rehabilitation”, as the 2012 WHO Discussion Paper clarifies [4], but focused on health care nonetheless. We therefore decided to limit the scope of our comparative normative analysis to the right to health care, although we acknowledge that the absence of broader health determinants within UHC does limit its ability to practically express the right to health in totality.

To be sure, our ambition was not to provide a complete account of the comparative advantages of two different norms – or two different sets of norms; we merely tried to find and expose the main differences.

### Comparing the right to health care with universal health coverage

#### The right to health care

The most authoritative formulation of the right to health is in article 12 of the International Covenant, which recognises “the right of everyone to the enjoyment of the highest attainable standard of physical and mental health” and prescribes specific steps for states to take in order to fully realise this right, including reducing the stillbirth rate and infant mortality; improving all aspects of environmental and industrial hygiene; preventing, treating, and controlling epidemic, endemic, occupational, and other diseases; and creating conditions that assure medical services and attention to all in the event of sickness [10].

The right to health has to be understood together with article 2(1) of the International Covenant, according to which states commit to “**take steps**, individually and through international assistance and co-operation, especially economic and technical, **to the maximum of its available resources**, with a view to achieving progressively the full realization of the rights recognized in the present Covenant by all appropriate means…” (emphasis added) [10]. Thus the right to health does *not* provide an immediate entitlement to the best available health care in the world. The corresponding obligation upon states is “to take steps towards” providing the best available health care, taking into account that states’ resources are limited and that there are many rights to be realised – even the wealthiest states do not have unlimited resources for health care. Due to the greater availability of resources, the level of health care that a government of a wealthy state is obliged to ensure its residents is broader than that which a government of a poorer state is obliged to ensure its residents, such that the entitlement to health care will look different across different countries.

The Committee, established in 1985 to carry out the monitoring functions foreseen in the International Covenant, not only issues comments on states’ progress reports; it also issues ‘general comments’ on specific issues, and in 2000 it issued General Comment 14 on “the right to the highest attainable standard of health” [11]. In General Comment 14, several important human rights principles are explained and applied to the right to health, including the following, which we identify for the purpose of comparison with UHC.

•First, the principle of *progressive realisation* demands of each state to use the maximum of its available resources, as they evolve over time. When unable to provide health care available in other parts of the world, states are obliged to demonstrate their inability [11]; paragraphs 9 & 47.

•Second, the principle of *non-discrimination* demands that the health care ensured by a given state to some people under its jurisdiction must be ensured to all people under its jurisdiction [11]; paragraphs 12, 18 & 19.

•Third, if states are not obliged to provide the best health care available in the world, and if they are not allowed do discriminate against any particular group, then how are they supposed to make choices between the health care they will provide and the health care they will not provide? The principle of non-discrimination implies the public health principle of *cost-effectiveness*. “Expensive curative health services which are often accessible only to a small, privileged fraction of the population, rather than primary and preventive health care benefiting a far larger part of the population”, have been qualified as “[i]nappropriate health resource allocation [that] can lead to discrimination that may not be overt” [11]; paragraph 19.

•Fourth, if the principle of non-discrimination implies a principle of cost-effectiveness, it also incorporates a principle of *participatory decision-making*. National public health strategies and plans of action that states are required to adopt and implement “shall be devised, and periodically reviewed, on the basis of a participatory and transparent process” [11]; paragraph 43f. Thus, determining the health care priorities is not purely a matter of epidemiology, but also of people’s expressed priorities.

•Fifth, “the process by which the strategy and plan of action are devised, as well as their content, shall give particular attention to all vulnerable or marginalized groups” [11]; paragraph 43f. Because of the principle of *prioritising vulnerable or marginalised groups*, even if a particular health concern affects only a small portion of the population – and might fall from consideration if a pure cost-effectiveness analysis guides decision-making – if it disproportionately affects vulnerable or marginalised populations, it may well be incumbent for the state to include it as part of the health care that it ensures for everyone.

•Sixth, all human rights have a minimum core, and all states, no matter how rich or poor, therefore have *minimum core obligations*. With regards to the entitlement to health care, states have “at least the following obligations: (a) To ensure the right of access to health facilities, goods and services on a non-discriminatory basis, especially for vulnerable or marginalized groups; (b) …; (c) …; (d) To provide essential drugs, as from time to time defined under the WHO Action Programme on Essential Drugs; (e) To ensure equitable distribution of all health facilities, goods and services;…” [11]; paragraph 43.

•Seventh, the right to health includes the principle of *shared responsibility*. Article 2(1) of the International Covenant on Economic, Social and Cultural Rights prescribes that states “take steps, **individually and through international assistance and co-operation**, especially economic and technical, to the maximum of its available resources, …” (emphasis added) [10], and when the Committee elaborated states’ core obligations arising from the right to health, it explicitly referred to international assistance: “For the avoidance of any doubt, the Committee wishes to emphasize that it is particularly incumbent on States parties and other actors in a position to assist, to provide ‘international assistance and cooperation, especially economic and technical’ which enable developing countries to fulfil their core and other obligations…” [11]; paragraph 45. Thus, states must prioritise these core aspects of health care in providing international assistance.

In short, under the right to health and the entitlement to health care in particular, people can expect access to health facilities that provide the essential medicines they may need. The level of health care they may expect is conditioned by the relative wealth of the countries they live in, but the health care that is made available to some inhabitants must be made available to all inhabitants, and they must be included in the decision-making processes that establish the standards. Furthermore, there is a minimum threshold, for which the international community – or states and other *in a position to assist* – must indeed provide assistance. This is particularly relevant for people living in low and lower middle income countries, where governments may be unable to fulfil their minimum core obligations without international assistance.

#### Universal health coverage

The 2010 World Health Report describes UHC as “access to good quality health services without people experiencing financial hardship because they must pay for care” [13]; page ix. Since the 2010 World Health Report, the concept of UHC has gained prominence and momentum, with numerous countries making commitments to achieve it [14], and the 2012 UNGA Resolution endorsing it [5]. But there is no single authoritative formulation of UHC.

The 2010 World Health Report refers to the 2005 WHA Resolution, which we consider as one of the authoritative formulations. The 2005 WHA Resolution urges member states:

(1) “to ensure that health-financing systems include a method for prepayment of financial contributions for health care, with a view to sharing risk among the population and avoiding catastrophic health-care expenditure and impoverishment of individuals as a result of seeking care;

(2) to ensure adequate and equitable distribution of good-quality health care infrastructures and human resources for health so that the insurees will receive equitable and good-quality health services according to the benefits package;

(3) to ensure that external funds for specific health programmes or activities are managed and organized in a way that contributes to the development of sustainable financing mechanisms for the health system as a whole;

(4) to plan the transition to universal coverage of their citizens so as to contribute to meeting the needs of the population for health care and improving its quality, to reducing poverty, to attaining internationally agreed development goals, including those contained in the United Nations Millennium Declaration, and to achieving health for all;

(5) to recognize that, when managing the transition to universal coverage, each option will need to be developed within the particular macroeconomic, sociocultural and political context of each country;

(6) to take advantage, where appropriate, of opportunities that exist for collaboration between public and private providers and health-financing organizations, under strong overall government stewardship;

(7) to share experiences on different methods of health financing, including the development of social health-insurance schemes, and private, public, and mixed schemes, with particular reference to the institutional mechanisms that are established to address the principal functions of the health-financing system” [12].

The 2010 World Health Report and the 2012 WHO Discussion Paper explain UHC in three ‘dimensions’.

•The first dimension is the extent of population covered under UHC. While universality ideally suggests that all people are covered, the 2010 World Health Report notes that “none of the high-income countries that are commonly said to have achieved universal coverage actually cover 100% of the population for 100% of the services available and for 100% of the cost – and with no waiting lists” [13], pages xv-xvi.

•The second dimension addresses the financial contribution covered by the government or government-supported schemes. This can include pooled funds, prepayment schemes, increased government spending and international assistance while trying to avoid *direct payments* by the patients – direct payments being “charges or fees are commonly levied for consultations with health professionals, medical or investigative procedures, medicines and other supplies, and for laboratory tests” [13], page 5. In this dimension of UHC, the ideal is that direct payments disappear, or if they have to be maintained, then it is at a level that does not exclude people from healthcare and does not create financial hardship. However, we must take into consideration both the different socio-economic settings and groups of and within each country in progressively achieving this ideal. Some countries may be able to do away with direct payments immediately, whereas others may be able to do away with them progressively.

•The third dimension of UHC as posited by WHO is the benefits in the health package. According to the 2012 WHO Discussion Paper, UHC implies that people “have access to *all* the services they need” (emphasis in original) [4]. The 2012 UNGA Resolution states that UHC “implies that all people have access, without discrimination, to nationally determined sets of the needed promotive, preventive, curative and rehabilitative basic health services” (emphasis added), and acknowledges that “when managing the transition of the health system to universal coverage, each option will need to be developed within the particular epidemiological, economic, socio-cultural, political and structural context of each country in accordance with the principle of national ownership” (emphasis added) [5]. The 2010 World Health Report, however, mentions that “[i]n lower-income countries, where prepayment structures may be underdeveloped or inefficient and where health needs are massive, there are many obstacles to raising sufficient funds through prepayment and pooling”, and that “[i]t is essential, therefore, that international donors lend their support” [13], page 6.

In short, under UHC, people could expect access to health facilities that provide the services they may need. As under the right to health care, the level of health care people may expect under UHC is conditioned by the relative wealth of the countries they live in – thus UHC tracks with the principle of *progressive realisation*. Also, UHC very explicitly aims to put an end to the discrimination that is caused by direct payments, and thus UHC affirms at least that element of the principle of *non-discrimination*. UHC seems to embrace the principle of *cost-effectiveness* as it promotes nationally determined sets of health services, developed within the epidemiological context of each country.

With regards to the principles of *participatory decision-making* and *prioritising vulnerable or marginalised groups*, UHC is less straightforward than the right to health care: the principle of national ownership advanced in the 2012 UNGA Resolution does not necessarily imply that the relevant decision-making processes will be participatory or prioritise vulnerable or marginalised groups, unless it is assumed that the national authorities duly represent the views of the people, including vulnerable and marginalised groups. The biggest difference, however, appears from comparing the right to health principles of *minimum core obligations* and *shared responsibility* with UHC. UHC – or at least the key documents we analysed to help explain the parameters of UHC – are rather ambiguous. While the 2010 World Health Report explicitly acknowledges the necessity of continued international assistance, the 2012 UNGA Resolution does not even mention international assistance among the sources of sustainable financing of UHC.

## Discussion

As mentioned above, the WHO considers UHC “a practical expression of the concern for **health equity and the right to health**” (emphasis in original) [4]. The concept of equity may help to understand and explain an essential difference between UHC – as it is being considered at present – and the right to health.

Equity is a concept to be handled with care. Equity refers to justice: the term *inequity* refers to “differences which are unnecessary and avoidable but, in addition, are also considered unfair and unjust” [15]. It takes a concept of justice to qualify measurable *inequalities* as *inequities*.

At the national level, there is a widely accepted concept of justice in health. In the 2005 WHA Resolution, it is formulated like this: “with a view to sharing risk among the population and avoiding catastrophic health-care expenditure and impoverishment of individuals as a result of seeking care” [12]. If inequalities between different population groups within the same country – like a difference in maternal mortality ratio between the poorest and the richest quintile of a population – can be attributed to insufficient ‘risk sharing’ – and if a result of this insufficient risk sharing is women not delivering their babies in health facilities due to costs they cannot easily afford – then the inequality is also an inequity.

But at the international or global level, there is no such widely accepted concept. If we would insert the word ‘global’ into the 2005 WHA resolution – which would then become “with a view to sharing risk among the global population” – than the huge health inequalities between countries could be qualified as inequities, because attributable, to some extent at least, to the lack of substantial cross-subsidies between wealthier and poorer countries that global risk sharing would imply. However, the ethical imperative of international transfers for health is not widely accepted.

International human rights law, and the right to health in particular, entails a somewhat conservative but nonetheless meaningful concept of global justice in health. The focus of international human rights law on obligations of states towards people under their jurisdiction, and its tolerance – acknowledgment without condemnation – of resource constraints that limit the practical implementation of state obligations, gives rise to a *state-centric* version of global justice that tracks rather well with *nationalist* interpretations of social justice. But its inclusion of *shared responsibility* – of the obligation “to take steps, individually and through international assistance and co-operation” – supports a certain level of *cosmopolitan* interpretations of global justice. While the extent of the obligation to provide assistance remains controversial, it covers at least minimum core obligations.

If the international community were to adopt UHC instead of the present MDG4, MDG5 and MDG6, then the post-MDG agenda would arguably move closer towards the right to health, if only because UHC promotes a comprehensive package of health care, while even the *sum* of efforts required to achieve MDG4 (child mortality), MDG5 (maternal health), and MDG6 (HIV/AIDS, malaria and other diseases), does not, for example, include treatment of non-communicable diseases for adults other than soon-to-be mothers. However, the 2005 WHA Resolution and the 2012 UNGA Resolution only briefly refer to international assistance, and where they do, they do not imply that sufficient international assistance ought to happen to ensure at least the basic level of UHC. The 2005 WHA Resolution mentions international assistance in its preamble – “Noting that some countries have recently been recipients of large inflows of external funding for health” – while the 2012 UNGA Resolution mentions “universal health coverage on the basis of solidarity at national and international levels” without clarifying the extent of expected solidarity at the international level. International assistance for UHC seems to be viewed as a windfall for some poorer countries, at the discretion of some wealthier countries – not something included in UHC per se. That may be a correct reflection of the present political reality, but is *not* a practical expression of the right to health, and it leaves global health inequalities standing as inequalities – not inequities. In low and lower middle income countries, UHC could mean access to a very cheap and incomplete package, not including antiretroviral AIDS treatment, for example.

However, UHC is a ‘work in progress’. In September 2013, the United Nations Sustainable Development Solutions Network (UNSDSN) presented a draft report on “Health in the Framework of Sustainable Development” for public consultation [16]. The UNSDSN proposes “Achieve Health and Wellbeing at All Ages” as the new health goal, and adds that “[t]o accomplish this objective we propose that all countries achieve universal health coverage at every stage of life, with particular emphasis on primary health services, including mental and reproductive health, to ensure that all people receive quality health services without suffering financial hardship”. Considering “Universal Health Care as being built on the foundation of human rights and equity”, the UNSDSN proposes a set of financing targets: public health care expenditure should be 3% of Gross Domestic Product (GDP) in low income countries; 3.5% of GDP in lower middle income countries; 4% of GDP in upper middle income countries; 5% of GDP in high income countries, and high income countries should furthermore allocate the equivalent of 0.1% of GDP to international assistance for health. According to our estimates, that would allow even the poorest countries to finance UHC at a level of US$55 per person per year. At present – estimates for 2010 – public health expenditure in low income countries is on average US$10 per person per year [17].

Would the equivalent of 0.1% of GDP of high income countries, shared in a reliable manner, make such a difference – a difference sufficient enough for the UNSDSN proposal to be considered a practical expression of the right to health care? As explained above, it would make a huge difference in low income countries. But for people who remember the Declaration of Alma-Ata [18], and who know that General Comment 14 was influenced by this declaration, 0.1% of GDP is like the “crumbs from the rich man’s table” [19]. The Declaration of Alma-Ata did not ask for more international assistance than 0.1% of GDP, on the contrary, it argued that primary health care should be at “a cost that the community and country can afford to maintain at every stage of their development in the spirit of self-reliance and self-determination” [18], section VI, i.e., without international assistance. The Declaration of Alma-Ata instead claimed a “New International Economic Order” [18], section III which would allow all countries to provide a decent level of health care. But the International Covenant and General Comment 14 do not go as far as the Declaration of Alma-Ata – even if General Comment 14 refers to it. Furthermore, even 0.1% of GDP, if provided in a reliable manner, could be the beginning of global redistribution, and thus contribute to a new international economic order. Therefore, we conclude that UHC, as proposed by the UNSDSN, can be called a practical expression of the right to health care.

## Abbreviations

2005 WHA Resolution: 2005 World Health Assembly resolution on “Sustainable health financing, universal coverage and social health insurance” [10]; 2010 World Health Report: World Health Report 2010 on UHC [11]; 2012 WHO Discussion Paper: WHO discussion paper on health in the post-2015 agenda [4]; 2012 UNGA Resolution: United Nations General Assembly resolution on “Global health and foreign policy”, adopted in December 2012 [5]; Committee: Committee on economic, social and cultural rights; GDP: Gross domestic product; International Covenant: International covenant on economic, social and cultural rights; MDGs: Millennium development goals; SDGs: Sustainable development goals; UDHR: Universal declaration of human rights; UHC: Universal health coverage; UNGA: United Nations general assembly; UNSDSN: United Nations sustainable development solutions network

## Competing interests

The authors declare that they have no competing interest.

## Authors’ contributions

GO and LAL conceptualised this paper and wrote the original draft. AW, CEB, RH, EAF, MM and LF participated in the comparative analysis and in revisions of the draft. All authors endorse the final paper. All authors read and approved the final manuscript.

## Pre-publication history

The pre-publication history for this paper can be accessed here:

http://www.biomedcentral.com/1472-698X/14/3/prepub
